# A Novel Optimized V-VLC Receiver Sensor Design Using μGA in Automotive Applications

**DOI:** 10.3390/s21237861

**Published:** 2021-11-26

**Authors:** Abrar Siddique, Tahesin Samira Delwar, Jee-Youl Ryu

**Affiliations:** Department of Smart Robot Convergence and Application Engineering, Pukyong National University, Busan 48513, Korea; abrarkhokhar.iiui@gmail.com (A.S.); samira.fset@gmail.com (T.S.D.)

**Keywords:** vehicular visible light communication, micro genetic algorithm, conventional genetic algorithm, offline computing

## Abstract

Vehicular visible light communication is known as a promising way of inter-vehicle communication. Vehicular VLC can ensure the significant advancement of safety and efficiency in traffic. It has disadvantages, such as unexpected glare on drivers in moving conditions, i.e., non-line-of-sight link at night. While designing a receiver, the most important factor is to ensure the optimal quality of the received signal. Within this context, to achieve an optimal communication quality, it is necessary to find the optimal maximum signal strength. Hereafter, a new receiver design is focused on in this paper at the circuit level, and a novel micro genetic algorithm is proposed to optimize the signal strength. The receiver can calculate the SNR, and it is possible to modify its structural design. The micro GA determines the alignment of the maximum signal strength at the receiver point rather than monitoring the signal strength for each angle. The results showed that the proposed scheme accurately estimates the alignment of the receiver, which gives the optimum signal strength. In comparison with the conventional GA, the micro GA results showed that the maximum received signal strength was improved by −1.7 dBm, −2.6 dBm for user Location 1 and user Location 2, respectively, which proves that the micro GA is more efficient. The execution time of the conventional GA was 7.1 s, while the micro GA showed 0.7 s. Furthermore, at a low SNR, the receiver showed robust communication for automotive applications.

## 1. Introduction

Visible light communication (VLC) has seen much interest in the field of wireless communication [1,2,3]. Visible light is the form of data communication from 375 nm to 780 nm [4]. The VLC communications approach is extremely advantageous. VLC has a wide bandwidth, which allows reaching very high data rates [5]. VLC can offer reliable secured communication because of the line-of-sight propagation [6]. Light-emitting diodes (LEDs) are commonly used as transmitters in most VLC systems. LEDs have great potentiality in terms of high performance, low cost, and efficiency. Besides, the semiconductor sector has improved LEDs’ performances, enabling the development of VLC systems. However, mostly, image sensors [7,8,9,10,11] or photodiodes (PDs) [12,13,14] are considered as the receivers. We also utilized an LED-PD in our proposed scheme.

### 1.1. Summary of the Recent Work

A prominent VLC application in the automotive industry is vehicle-to-vehicle (V2V) communication, which is compulsory to increase the efficiency and safety of vehicles and contributes to the enhancement of road traffic regulations and estimations. Smart transport infrastructure development is a crucial future challenge. Due to the high-cost performance, the VLC system is suitable for V2V or V2I/I2V communication. In the outdoor V2V or V2I/I2V scenario, the major problem is the noisy VLC channels [15,16,17]. Several noise sources and numerous disturbing aspects are responsible for the problems with the vehicular VLC channel. Furthermore, the communication distance is much larger in contrast with the indoor links, and the received signal’s optical radiance consequently decreases to tens of nW/cm^2^, which affects the SNR. Additionally, the distances between the transmitter (TX) and receiver (RX) vary, which changes the SNR level, making the channel unpredictable and dynamic, since the cars are in continuous motion. However, this is challenging to ensure, because the outside VLC channel includes several noise sources, high mobility levels, varying communication distances, and unexpected conditions. The influences of the noise and additional light causes the problem in outdoor VLC communications. The above problems were shown in [18], where it was mentioned that, due to the artificial light sources, the outdoor VLC channel is strongly affected. The authors also explained how parasitic light can saturate the receiver and, thus, impede communication. Therefore, to solve this issue, many researchers have proposed different types of approaches, using narrow-angle receivers [19] and optical filters [20] at the receiver side. Other prior research also considered the NLOS link in the VLC scenario. For example, in [21], a channel characterization investigation was performed, and it showed three scenarios for the PD alignment of the receiver, in the case of an NLOS link. In [22], various configurations of the channel characterization, experimented together with the path loss and impulse response, were proposed. The characteristics of wireless channels (indoor) and their communication efficiency were highlighted in these investigations. In [23], the receiver alignment was presented in a tilting approach to obtain the maximum optical power. However, the limitations of [23] were that this was discussed only for the LOS link, while the NLOS link was ignored. Besides, in the case of a practical scenario, this scheme failed to show the rotation of the PD. The authors applied a traditional GA to optimize the signal strength in indoor VLC scenarios [24,25]. In another recent work [26], a parallel evolutionary artificial potential field was applied to achieve the optimal path considering the complex real-world scenario. In [27], the membrane evolutionary artificial potential field (memEAPF) method was implemented to solve MR path planning problems using the GA.

### 1.2. Motivations

**Figure 1:**
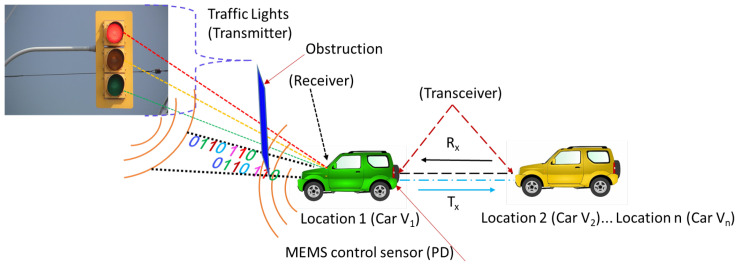
An outdoor configuration of several vehicles (VLC).

One of the main considerations in the design of a V-VLC receiver is the optimum quality of a received signal and a low SNR. The SNR is the amount indicated by the signal strength relative to that signal’s noise power. Mostly, V-VLC has drawbacks for drivers in moving conditions (NLOS link), as it consists of light paths through reflections. To address these issues, we demonstrate a circuit-level receiver design for VLC outdoor applications in this paper. Then, we applied a scheme in the proposed receiver for optimizing the received signal strength (RSS) using the micro GA (μGA). The outdoor VLC system’s configuration of several vehicles is shown in Figure 1. An outdoor configuration is presented where traffic LEDs act as the transmitters and PDs act as the receivers and measure the RSS.

In this scenario, the RX of the neighboring vehicles Car v_1_ and Car v_2_ using the traffic lighting systems can detect the RSS_max_ using the μGA. In addition, this study shows how the user can redirect the device to the ideal alignment to receive the RSS_max_. It was designed in particular to communicate in night conditions. This paper shows a μGA-based optimization to compute the optimum PD alignment for a real NLOS V2V system. Before the transmission, the μGA’s optimization was performed, and the determined optimum PD alignment was therefore helpful to rotate the PD. To determine the PD alignment, considering the NLOS environment, the parameters were measured, i.e., the angle-of-arrival of the light that converges on the PD. The amplitude of the individual RSS is the intensity of the light. For the ease of the driver, while driving at night, it is obvious that the proposed scheme would help the driver achieve the optimal communication quality and maximum RSS, thus showing the driver a possible direct solution of the RSS_max_, where at the final stage, the PD could be directed with the help of MEMS and find an optimum angle to move the vehicle forward in the correct direction. As per the authors’ knowledge, the proposed scheme has not been studied in V-VLC systems. The findings obtained are highly promising, and the approach presented is suited for automotive applications; even in low SNR situations, it achieved good BER values.

### 1.3. Our Contributions

The contributions of this paper are as follows:

1. To enhance vehicular VLC systems with self-aware capabilities, which would maximize the communication performances and efficiency, we present a novel optimized receiver designed for automotive applications;
2. We show the circuit design of the receiver and implemented a micro genetic algorithm (i.e, meta-heuristic searching algorithm) to optimize the maximum received signal strength to ensure the best communication quality in V-VLC. Besides, our proposed algorithm can dictate the alignment of the receiver instead of measuring the signal for each angle;
3. To provide a clear insight into our proposed algorithm, we analyzed the characteristics and optimization factor of the chosen μGA;
4. We compared and analyzed the accuracy and the efficiency of the chosen μGA over the conventional genetic algorithm;
5. To solve complex real-world problems, we discuss the challenges and future directions of using the evolutionary algorithm, which can provide a reference framework for future research.

In a nutshell, a novel optimized V-VLC receiver design is proposed, which can optimize the RSS_max_ for V2V communication and can provide a low SNR. The rest of the paper is as follows. The design of the proposed V-VLC receiver is shown in Section 2. In Section 3, the results are described. Finally, in Section 4, the conclusion and future research works are discussed.

## 2. Methods and Materials

The work in this research includes the circuit design of the RX and the implementation of a μGA to optimize the RSS for the V-VLC.

### 2.1. Design of the Proposed V-VLC Receiver

**Figure 2:**
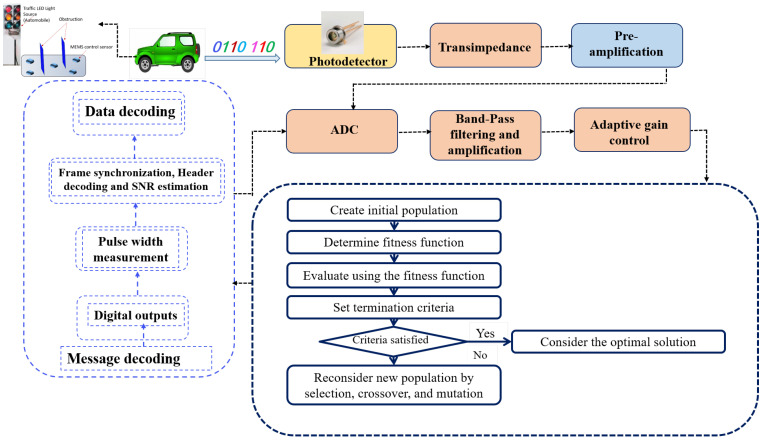
Proposed V-VLC receiver design.

The proposed V-VLC receiver’s conceptual setup is shown in Figure 2. The proposed V-VLC receiver concept was based on a PIN photodiode photosensitive element.

The proposed scheme addresses several significant adaptability issues. In automotive applications, a higher data rate is preferable. Thus, the proposed V-VLC receiver design was intended to receive and correctly decode messages transmitted at various data rates. The V-VLC RX also included μGA-based optimization. We used the μGA to optimize the RSS in the V-VLC RX. After being reflected from the various walls, the NLOS links consist of many pathways from the TX to the PD. Analyzing all the possible paths, the method of computing the optimum path is termed optimization. During the optimization process, difficult problems cannot be resolved by utilizing a gradient method; however, these can be solved by multi-modal optimization [28]. The non-gradient approaches, such as the CGA or μGA, are therefore required [29,30]. The motivation for utilizing the μGA and CGA is clear, because they allow optimum outcomes to be produced with great precision, although taking a complicated and extensive space for assumptions into account [31]. In the current research, we dealt with a huge number of coordinates so that there were also more I/P and O/P combinations. In a practical NLOS VLC vehicular system, the proposed method computes the best suitable alignment of the PD. The proposed scheme deals with an off-line computation. Optimization of the μGA took into account many converging PD light beams during the process. The convergence to an optimal RSS, i.e., the condition that meets the criteria for optimization, was validated in two different locations (Car V_1_ and Car V_2_). Before the transmission, the μGA’s optimization took place, and hence, the optimal alignment calculated for the PD is convenient for the direct rotation of the PD.

### 2.2. System Circuit Diagram

**Figure 3:**
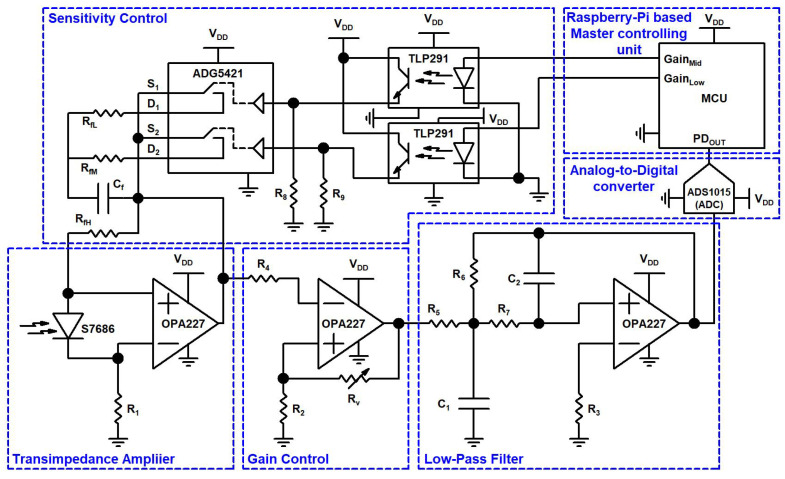
Circuit diagram of the V-VLC RX design.

**Figure 4:**
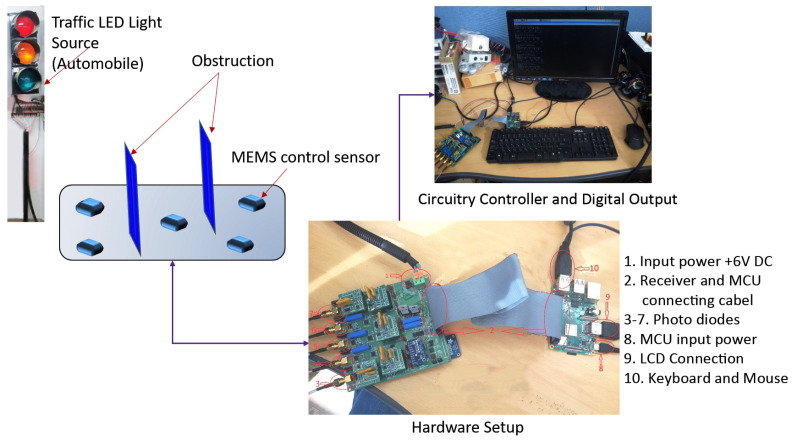
Proposed V-VLC receiver design’s hardware setup.

The schematic of the RX circuit and its hardware setup are shown in Figure 3 and Figure 4, respectively. The receiver design consisted of a light-collecting unit that incorporates a globe, a high-precision luminosity filter, a transimpedance amplifier (TIA), a gain controller (GC), a low-pass filter (LPF), an analog-to-digital converter (ADC), a master controlling unit (MCU), and a sensitivity control circuit (SC). The TIA, GC, and LPF use a precision amplifier (OP-AMP), the OPA227 (Texas Instruments, Dallas, TX, USA). This OP-AMP has a low noise of 3 nV/(Hz)1/2, a high speed with a slew rate of 2.3 V/us, and a high open loop gain of 160 dB. After passing through the globe and high-precision luminosity filter, incident light strikes the light sensor, which generates current according to the human eye’s visual perception. This current is minuscule, on the order of several pico-amperes for low-illuminance values or several dozen micro-amperes for high-illuminance values. The sensor used in our design was the S7686 silicon photodiode manufactured by HAMAMATSU. The S7686’s spectral response characteristics are similar to the human eye’s sensitivity and also analogous to Commission Internationale de l’Eclairage’s (CIE) spectral luminous efficiency. The electrical and optical characteristics of the S7686 are given in Table 1.

The output current of the S7686 is converted into a voltage signal by a TIA. The current generated by the S7686 sensor flows to the output side of the TIA, via a feedback resistance. This current is I and the feedback resistance is R, so the voltage given by (I × R) characterizes the amplifier’s output. This current is proportional to the incident light at the S7686 sensor. The feedback resistance generates voltages at the output node of the I-V amplifier. The minimum value of these voltages is approximately 0 V in the presence of dark current, while the maximum value of these voltages is 6 V, equal to the positive input voltage supply in the presence of the saturation current of the S7686 sensor. Despite the minuscule magnitude of the S7686 sensor’s current and the different values of incident light on the sensor, the conversion to a suitable voltage level was handled using feedback resistance switching using an SC circuit block. The TIA output is amplified by the GC circuit. The TIA was implemented to achieve stable measurement at low-illuminance levels. As the input signal can have variable intensity, the GC circuit provides an output signal with a constant amplitude.

The different values of the feedback resistance are offered to the TIA to set the measuring range and sensitivity range of the illuminance and the control signal level from the MCU with different resistive combinations, the measuring range, and the output of the TIA, GC, and LPF, given below in Table 2. The feedback resistance depends on the different resistive combination values of resistors R_fH_, R_fM_, and R_fL_. These resistive combinations are controlled by the MCU using control signals, utilizing an analog switch (AD5421) and photo coupler (TLP291), given in the table below.

Next, the signal is digitalized using an analog-to-digital converter (ADC). At this level, the ADC sampling rate and the ADC resolution are important factors that determine the performance of the system. Therefore, the sampling frequency will significantly influence the filtering process and the signal processing quality, while determining the computational power requirement. Therefore, the ADC sampling frequency should be established based on a trade-off between the performances and the available computational resources. In the considered model, the signal provided by the transimpedance circuit was sampled at a resolution of 0.008 V, corresponding to a 12 bit ADC resolution for a 3.3 V input. A higher sampling frequency can significantly improve the quality of the filtering and, as consequence, the system performances.

**Figure 5:**
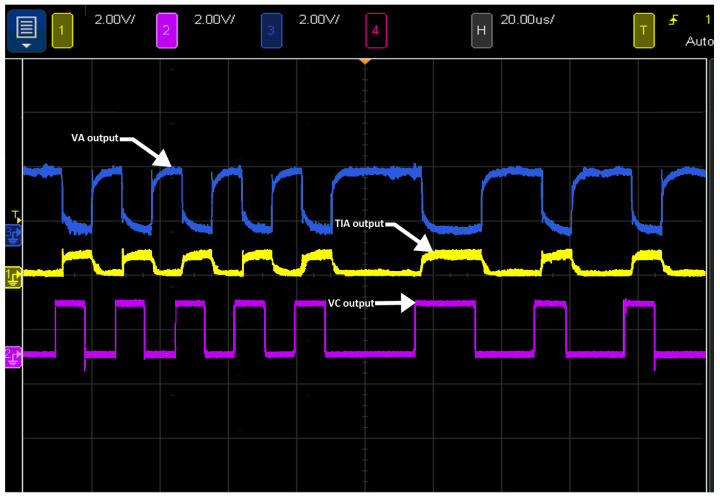
Output waveforms of the TIA, GC, and LPF.

In the proposed receiver design, the signal output after each stage was observed with the DSO7054A oscilloscope. As shown in Figure 5, the TIA output signal consists of a rectangular wave signal, and it is affected by noise. It is shown that the GC output signal is amplified, and the LPF output signal removes the low-frequency components and noise from the GC output signal.

### 2.3. Problem Formulation

**Figure 6:**
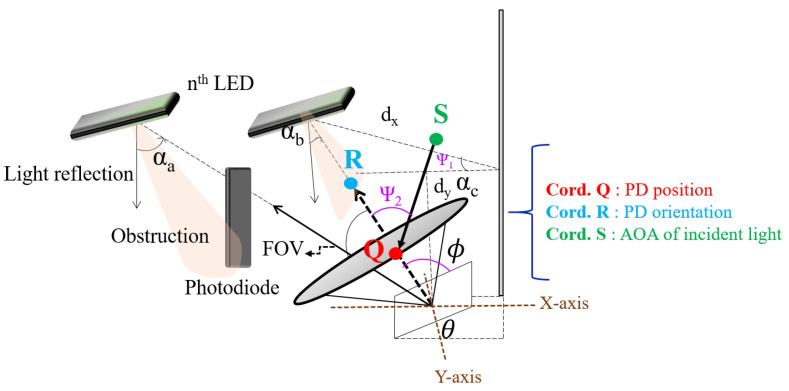
Graphical representation of the proposed receiver coordinates.

In our scheme, as we considered the NLOS links only, the communication can be possible by means of signals coming through reflections from different sides of the walls. Each reflector has a particular spectral reflectance, and the reflectivity range varies as the wavelength changes [32]. The system diagram of the NLOS environment considered in the proposed scheme is given. In our present work, we focused on a single reflection, which is depicted in Figure 6, and it shows the graphical representation of the proposed receiver coordinates [24].

The channel’s response after the first reflection [21] is,

(1) $$h^{\left(1\right)}\left(t,\Phi_{n}\right)=\int_{s}L_{1}L_{2}\Gamma_{n}^{\left(1\right)}rect\left(\frac{\psi_{2}}{FOV}\right)\times \delta \left(t-\frac{d_{x}+d_{y}}{C}\right)dA_{ref}$$

where:

(2) $$L_{1}=\frac{A_{ref}\left(m+1\right)\cos^{m}\psi_{1}\cos\alpha_{a}}{2\pi d_{x}^{2}}\;$$

(3) $$L_{2}=\frac{A_{\mathrm{PD}}\cos\psi_{2}\cos\alpha_{b}}{\pi d_{y}^{2}}$$

Equation (1) considers the entire surface of the wall, i.e., the reflector:

*A*_*ref*_—reflector area;
*A*_PD_—the area of the PD;
*C*—speed of light;
*FOV*—field of view of the PD.

The light having an incident angle ψ_2_ less than or equal to the *FOV* is detectable by the PD. The rectangular function in Equation (1) is given by [21]:

Rec(x) = 1, when |*x*| ≤ 1;
Rec(x) = 0, when |*x*| > 1.

The power attained from the first reflection can be written as,

(4) $$\Gamma_{n}^{\left(1\right)}=\int_{\omega }\Phi_{n}\rho_{1}\left(\omega \right)d\omega \;$$

Here, ρ_1_(ω) is the reflector’s spectral reflectance. The optical signal power that is received by the PD is given by,

(5) $$P_{r}=H\left(0\right)P_{t}$$

*H*(0) is the channel DC gain, and *P_t_* is the transmitted power. Therefore, we can describe the PD photocurrent as [21],

(6) γ(t)=RX(t)⊗h(t)+N(t)

Here:

X(t)—transmitted optical pulse;
N(t)—noise;
h(t)—power delay product (PDP);
*R*—responsivity.

### 2.4. Proposed μGA and Its Advantages over the CGA

Evolutionary algorithms (EAs) are effective heuristic search methods based on Darwinian evolution with strong robustness and flexibility [33,34,35]. EAs are useful to find the optimum solution at the beginning of the optimization process. One of the prominent classes of EAs is the CGAs, which follow the principle of evolution in nature [36,37]. The CGA is a powerful algorithm, and it is used to solve complex problems. Regardless of the several advantages of the CGA, it also has some shortcomings, as it requires a large set of solutions to converge at an optimum value through a repetitive process, and it consumes much processing time and many resources. Due to this issue, the CGA might result in some complications for applications where the time parameter is critical. The μGA is a variant of the CGA. The μGA is a very straightforward, yet powerful way of solving the most complicated problems more quickly than other heuristic methods. As compared to the CGAs, the μGA is much faster [38,39]. The μGA provides optimal solutions without having to estimate additional parameter inputs such as the rate of mutation. The optimization speed is quicker in the case of the μGA, as each generation has fewer function evaluations than the traditional CGAs. The reason for choosing the μGA over the traditional CGAs is the smaller population size, instead of a bigger population size, as other heuristic methods [39]. The μGA provides some advantages over the CGA, for example the simplicity in the design and less processing time.

**Figure 7:**
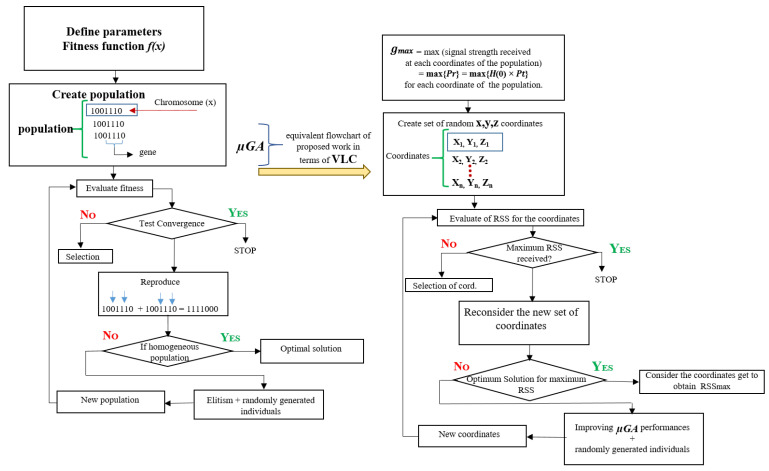
Algorithm of the proposed μGA-based V-VLC receiver.

Figure 7 shows the proposed μGA algorithm for the V-VLC receiver. In the initial stage, the μGA generates a set of 50 coordinates. Each coordinate indicates a specific location in the target place. Then, the coordinates R are computed to obtain the RSS. With the help of the crossover operation, the population of 50 coordinates is able to generate another population of 50 coordinates. In the new population, the RSS for all these new 50 coordinates is considered. From the total of 100 coordinates (population size), the selection procedure chooses only the 50 coordinates that have the highest RSS. In the next step, the others are rejected. The 50 coordinates chosen by a CGA participate with a mutation rate of 0.01, with re-calculations of the signals received. Thus, a whole generation is complete at this point. This procedure goes on until the ideal solution is reached. The current RSS is compared to the previous RSS by the μGA. Next, the current and past values’ difference can help optimize the RSS.

### 2.5. Optimization Factors

**Figure 8:**
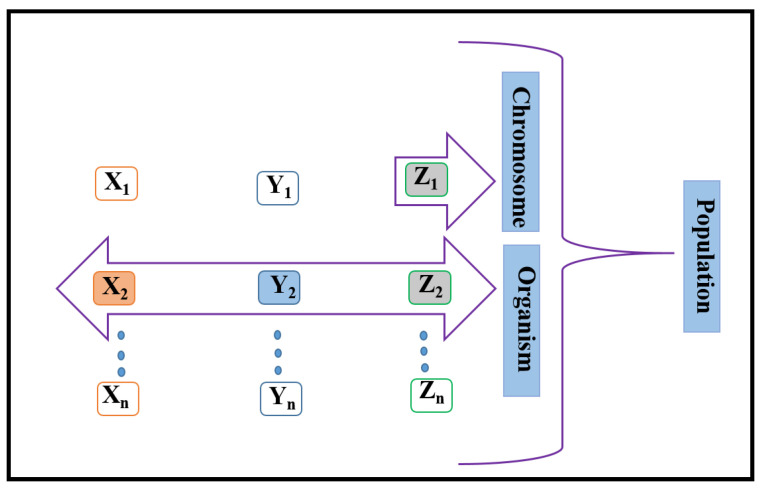
A simple diagram of the μGA’s optimization factors.

Figure 8 describes the simple diagram of the μGA’s optimization factors. In the initial stage, our proposed μGA generates a small number of coordinates (i.e., a minimum population of chromosomes). A complete combination of chromosomes is called an organism. In our work, organism refers to the coordinates of the PD location, i.e., *X_N_*, *Y_N_*, *Z_N_*. A set of organisms is known as a population; this refers to a collection of coordinates. As our work deals with signal strength maximization, the fitness function we used is, *g*_max_ = *RSS*_max_, at individual coordinates of the population) = max{*P_r_*}= max{*H*(0) × *P_t_*} for individual coordinates of the population. The fitness of the coordinates (*X_i_*, *Y_i_*, *Z_i_*) = the power received from the coordinates (*X_i_*, *Y_i_*, *Z_i_*). The objective function (or fitness function) = max [power received from (*X*_1_, *Y*_1_, *Z*_1_), (*X*_2_, *Y*_2_, *Z*_2_), … (*X_N_*, *Y_N_*, *Z_N_*) coordinates], where *N* is the population size (or the number of coordinates).

In the process of any individual iteration, a chromosome from the present population will be thoroughly tested with the two genetic operators, mutation and crossover. This process aims to generate good offspring. In our work, we considered the *X* coordinate, *Y* coordinate, and *Z* coordinate, which can be referred to as chromosomes. The other optimization factor is crossover. In this process, the population can produce the next offspring. The offspring reproduction process is the same as the reproduction process of humans. In this reproduction process, the DNA of a child comprises half the DNA of the parents. Here, the *X*, *Y*, and *Z* coordinates are swapped to produce the next offspring. For example, the offspring may be (*X*_1_, *Y*_1_, *Z*_1_), (*X*_2_, *Y*_2_, *Z*_2_), (*X*_3_, *Y*_3_, *Z*_3_) (*X*_4_, *Y*_4_, *Z*_4_)…(*X_N_*, *Y_N_*, *Z_N_*) coordinates]. Mutation reduces the search time by obtaining different solutions to converge quickly. Only a few *X*, *Y*, and *Z* coordinates are randomly altered throughout the mutation process during offline computing. The original chromosome will be replaced by the offspring when a better offspring is generated. This is performed until mostof the chromosomes have come to a similar solution or the supply bounds are surpassed, for instance the number of iterations. To make things easier and efficient, the best chromosomes in the current population stay only after their fitness function has been classified, which is then improved opportunistically by both genetic operators across consecutive generations. Elitism means an improvement of the μGA’s performance. The primary goal is to pass on the best of the current generation’s entities to the next. Sometimes, during the crossover or mutation, a potentially optimal candidate may be lost. The μGA can revive the lost candidate with elitism in the following generations. Elitism is the practice of copying the smallest proportion of the best-fitting candidate for future generations. It offers a significant role in the performance to ensure that the μGA does not waste time rediscovering incomplete solutions previously abandoned.

## 3. Results and Discussion

This section is intended to sum up the premises for the simulation, describe the results and findings, and provide a short overview of the performance of the V-VLC receiver scheme. A review of the published state-of-the-art in VLC (in terms of the RSS) is shown in Table 3.

We carried out simulations with LED transmitters, a user with two different locations, and obstacles in the target place. Table 4 describes the key simulation components of the NLOS environment, and Table 5 and Table 6 show the parameters utilized for the CGA and μGA, respectively.

The parameters of the CGA and μGA have a distinct and diverse effect on the output results. The probabilities of these parameters affect the output of the overall systems, i.e., for the crossover, a 100% success ratio has a different impact compared to a 50% success ratio. The same scenario applies to the probability of mutation. The mutation and crossover rate balancing is an important aspect in the CGA and μGA. The final results, quality, and speed could be affected by the population size. However, in our case, to achieve an optimal result, we chose the mutation rate for the μGA of 0.01, when the population size was 30 individuals, because a high mutation rate could lead the search to be random.

**Figure 9:**
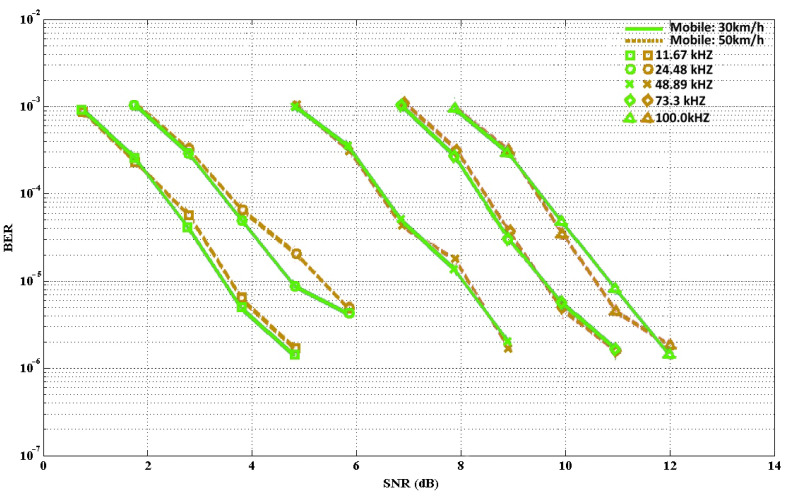
BER for several frequencies (mobile conditions).

**Figure 10:**
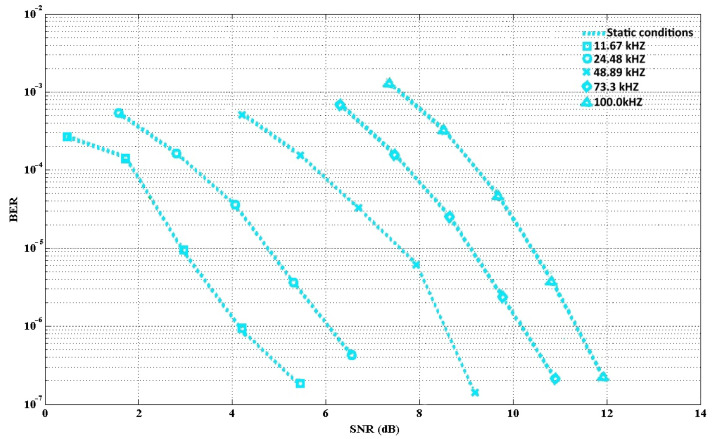
BER for several frequencies (static conditions).

The MATLAB tool was utilized to perform the simulation. Figure 9 and Figure 10 describe the BER at various velocities in mobile and static conditions respectively. Considering the five modulation frequencies, the BER results are shown. Figure 9 shows, in the case of the mobile condition, that the SNR increased; thus, errors occurred. As a result, this caused an increasing gap between the static and mobile conditions. Figure 10 shows that the simulations were performed under static conditions with BERs ranging from 10^−3^–10^−7^. However, in the case of lower frequencies, the increment of the BER was not very strong, and it was more sensitive to the noise in the case of higher frequencies.

### 3.1. RSS_max_ for PD Location 1 (without CGA and μGA Optimization)

**Figure 11:**
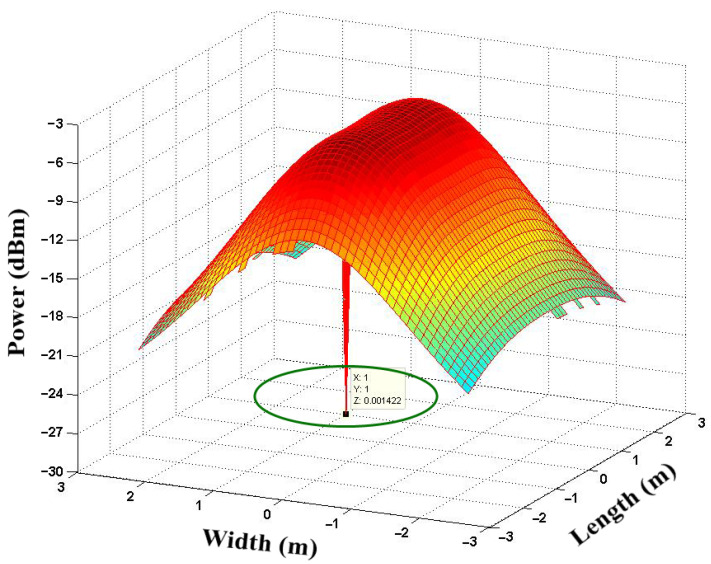
Allocation of the signal power received at RX Location 1 (1,1,0).

Figure 11 shows the allocation of the signal power received in RX Location 1, (1, 1, 0). The result shows that the received signal power was equal to −26.50 dBm at the (1, 1, 0) user location without any optimization process.

#### 3.1.1. RSS_max_ for PD Location 1 (with CGA and μGA Optimization)

To achieve the RSS_max_, we performed cost minimization in this work. Thus, in the μGA’s minimization, we chose the objective by selecting each entity with the best fitness, i.e., the lowest fitness values.

**Figure 12:**
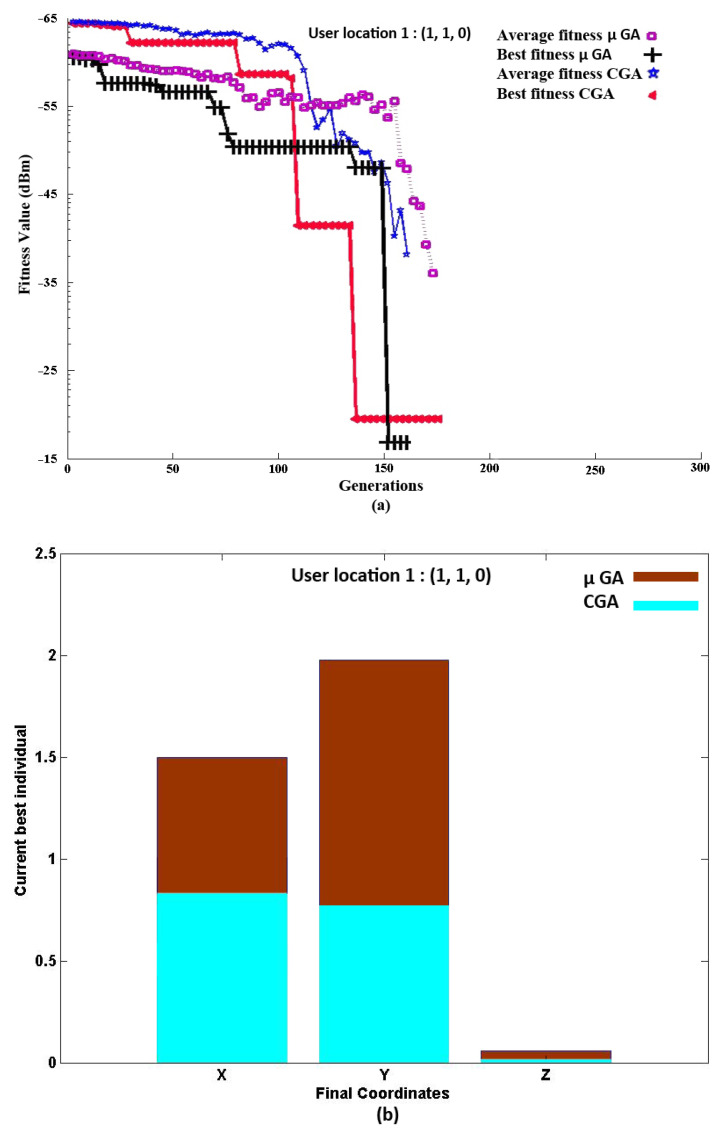
RX Location 1, (1, 1, 0): (**a**) Convergence of the CGA and μGA to the RSS_max_. (**b**) Final coordinates of the CGA and μGA.

We can observe from Figure 12a for PD Location 1 the convergence of the CGA and μGA to the RSS_max_, i.e., the best fitness: −19.6 dBm (for the CGA) and −17.9 dBm (for the μGA), respectively. From the simulation results, it can be shown that in the beginning of the CGA and μGA, both could not achieve a higher RSS. However, over time, while new generations were developed, both algorithms were able to find the final coordinates for the PD alignment to achieve the maximum RSS. The result showed at the 174th and 154th generation that the CGA and μGA converged to their optimum alignment. The final coordinates *R*’s global maxima for the CGA and μGA are shown in Figure 12b. The *X*, *Y*, and *Z* variables represent the PD length, width, and height as well. The final coordinates of the CGA are (*X*, *Y*, *Z*: 0.8 m, 0.7 m, 0.2 m) and of the μGA are (*X*, *Y*, *Z*: 1.5 m, 1.9 m, 0.4 m), respectively. According to the figure, the final coordinates *R*_1_ are the combination of the three variables (*X*, *Y*, *Z*) that determine the final location of the PD. Thus, in this way, the PD should be aligned towards the RX to obtain the maximum RSS. In the case of the CGA and μGA, the the signal power received of −26.50 dBm (without optimization) was improved to −19.6 dBm (with optimization) and of −26.50 dBm (without optimization) was improved to −17.9 dBm (with optimization), demonstrating that the proposed algorithm improved the communication quality in the NLOS indoor VLC scenario and could be compared fairly.

#### 3.1.2. Finding the Alignment of the RSS_max_ at User Position 1

The alignment of finding the RSS_max_ is reflected in Table 7.

The azimuth and elevation angle can be written for the CGA as,

(7) $$\theta =\tan^{-1}\left(\frac{Y-Rx_{y}}{X-Rx_{x}}\right)=\tan^{-1}\left(\frac{0.7-1}{0.8-1}\right)=56.30^{\circ }$$

(8) $$\begin{array}{c} \phi =\tan^{-1}\left(\frac{\sqrt{{\left(X-Rx_{x}\right)}^{2}+{\left(Y-Rx_{y}\right)}^{2}}}{Z}\right)\\ =\tan^{-1}\left(\frac{\sqrt{{\left(0.8-1\right)}^{2}+{\left(0.7-1\right)}^{2}}}{0.2}\right)\\ =60.98^{\circ } \end{array}$$

The azimuth and elevation angle can be written for the μGA as,

(9) $$\theta =\tan^{-1}\left(\frac{Y-Rx_{y}}{X-Rx_{x}}\right)=\tan^{-1}\left(\frac{1.9-1}{1.5-1}\right)=60.94^{\circ }$$

(10) $$\begin{array}{c} \phi =\tan^{-1}\left(\frac{\sqrt{{\left(X-Rx_{x}\right)}^{2}+{\left(Y-Rx_{y}\right)}^{2}}}{Z}\right)\\ =\tan^{-1}\left(\frac{\sqrt{{\left(1.5-1\right)}^{2}+{\left(1.9-1\right)}^{2}}}{0.4}\right)\\ =68.58^{\circ } \end{array}$$

*Rx_x_*—RX location in the *X*-coordinate; *Rx_y_*—RX location in the *Y*-coordinate; *Rx_z_*—RX location in the *Z*-coordinate.

Additionally, *X*, *Y*, and *Z* represent the space where the PD will be directed for the RSS_max_. The μGA’s and CGA’s final solution yielded these *X*, *Y*, and *Z* (coordinates). As a result, determining the azimuth and elevation angles assisted the MEMS-controlled PD in rotating in the alignment of the maximum signal strength in NLOS indoor VLC.

### 3.2. RSS_max_ for PD Location 2 (without CGA and μGA Optimization)

**Figure 13:**
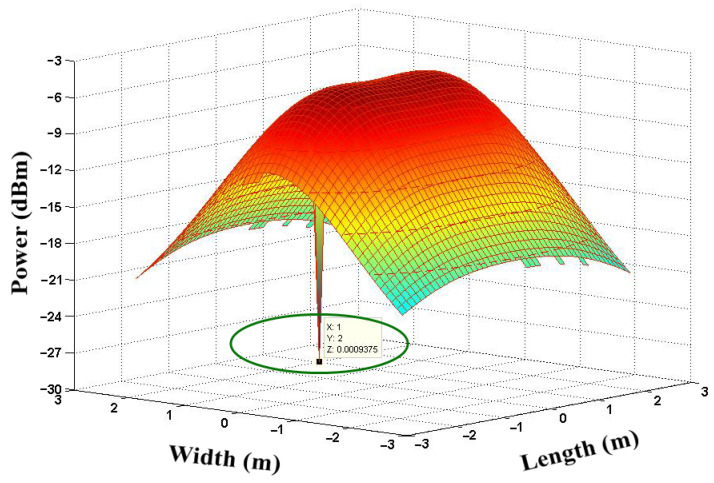
Allocation of the signal power received at RX Location 2, (1,2,0).

The allocation of RX power in NLOS links is shown in Figure 13 with an obstruction. We considered RX Location 2, (1,2,0), where we found, without optimization, that the RX signal power was −28.46 dBm.

#### 3.2.1. RSS_max_ for PD Location 2 (with CGA and μGA Optimization)

**Figure 14:**
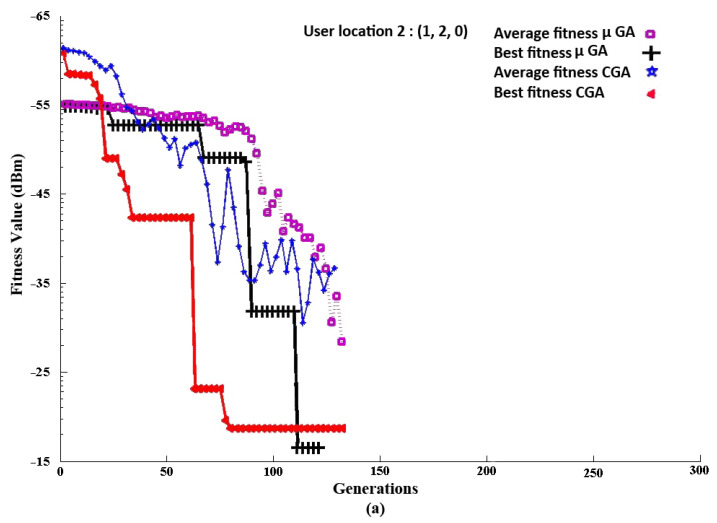

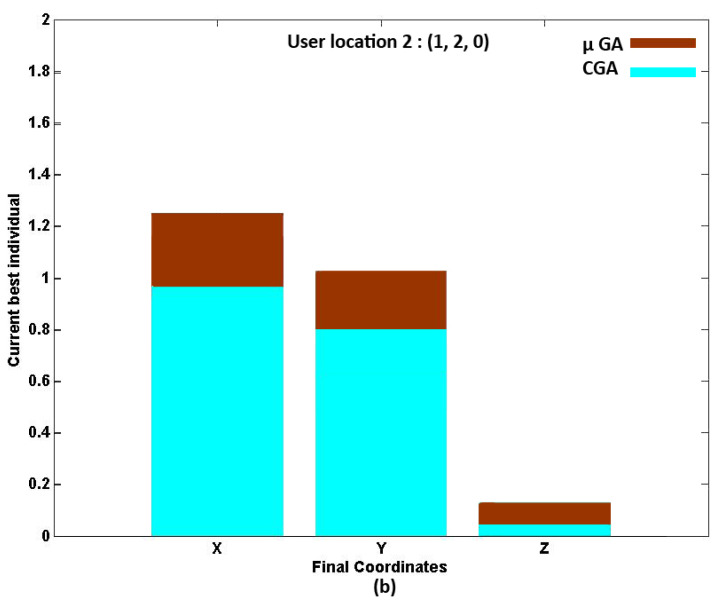
RX Location 2, (1,2,0): (**a**) Convergence of the CGA and μGA to the RSS_max_. (**b**) Final coordinates of the CGA and μGA.

An additional receiver location was simulated to justify and demonstrate the efficacy of the proposed scheme. The same as before, in Figure 14a, for Location 2, i.e., (1, 2, 0), the convergence curves of the CGA and μGA are shown. The RSS_max_ in this location for the CGA was −14.7 dBm, and it converged at the 127th generation; for the μGA, it was −12.1 dBm and converged at the 124th generation. The coordinates *R*_2_ for the CGA and μGA are (0.9 m, 0.8 m, 0.08 m) and (1.3 m, 1.0 m, 0.1 m), shown in Figure 14b, for the alignment of the PD.

#### 3.2.2. Finding the Alignment of the RSS_max_ for User Position 2

Finding the alignment of the RSS_max_ is reflected in Table 8.

The azimuth and elevation angle can be written for the CGA as,

(11) $$\theta =\tan^{-1}\left(\frac{Y-Rx_{y}}{X-Rx_{x}}\right)=\tan^{-1}\left(\frac{0.8-2}{0.9-1}\right)=85.23^{\circ }$$

(12) $$\begin{array}{c} \phi =\tan^{-1}\left(\frac{\sqrt{{\left(X-Rx_{x}\right)}^{2}+{\left(Y-Rx_{y}\right)}^{2}}}{Z}\right)\\ =\tan^{-1}\left(\frac{\sqrt{{\left(0.9-1\right)}^{2}+{\left(0.8-2\right)}^{2}}}{0.08}\right)\\ =86.19^{\circ } \end{array}$$

The azimuth and elevation angle can be written for the μGA as,

(13) $$\theta =\tan^{-1}\left(\frac{Y-Rx_{y}}{X-Rx_{x}}\right)=\tan^{-1}\left(\frac{1-2}{1.3-1}\right)=-73.30^{\circ }$$

(14) $$\begin{array}{c} \phi =\tan^{-1}\left(\frac{\sqrt{{\left(X-Rx_{x}\right)}^{2}+{\left(Y-Rx_{y}\right)}^{2}}}{Z}\right)\\ =\tan^{-1}\left(\frac{\sqrt{{\left(1.3-1\right)}^{2}+{\left(1-2\right)}^{2}}}{0.1}\right)\\ =84.52^{\circ } \end{array}$$

#### 3.2.3. Benchmark Testing and Holm–Bonferroni Statistical Test

The ten computationally benchmark functions shown below are utilized to justify the performance of the proposed optimization algorithm.

(15) $$F_{1}\left(X\right)=\Sigma_{i=1}^{n}x_{i}^{2};{\left[-100,100\right]}^{n}$$

(16) $$F_{2}\left(X\right)=\Sigma_{i=1}\left|x_{i}\right|+\Pi_{i=1}^{n}\left|x_{i}\right|;{\left[-10,10\right]}^{n}$$

(17) $$F_{3}\left(X\right)=\Sigma_{i=1}{\left(\Sigma_{j}^{i}x_{j}\right)}^{2};{\left[-100,100\right]}^{n}$$

(18) $$F_{4}\left(X\right){=}_{i}^{max}\left\{\left|x_{i}\right|,1\leq i\leq n\right\};{\left[-100,100\right]}^{n}$$

(19) $$F_{5}\left(X\right)=\Sigma_{i=1}^{n-1}\left[100{\left(X_{i+1}-x_{i}^{2}\right)}^{2}+{\left(x_{i}-1\right)}^{2}\right];{\left[-30,30\right]}^{n}$$

(20) $$F_{6}\left(X\right)=\Sigma_{i=1}^{n}\left[x_{i}+0.5^{2}\right];{\left[-100,100\right]}^{n}$$

(21) $$F_{7}\left(X\right)=\Sigma_{i=1}^{n}ix_{i}^{4}+random\left[0,1\right];{\left[-1.28,1.28\right]}^{n}$$

(22) $$F_{8}\left(X\right)=\Sigma_{i=1}^{n}-x_{i}sin\left(\sqrt{\left|x_{i}\right|}\right);{\left[-500,500\right]}^{n}$$

(23) $$F_{9}\left(X\right)=\Sigma_{i=1}^{n}\left[x_{i}^{2}-10cos\left(2\pi x_{i}\right)+10\right];{\left[-5.12,5.12\right]}^{n}$$

(24) $$F_{10}\left(X\right)=-20exp\left(-0.2\sqrt{\frac{1}{n}\Sigma_{i=1}^{n}x_{i}^{2}}-exp\left(\frac{1}{n}\Sigma_{i=1}^{n}cos\left(2\pi x_{i}\right)\right)+20+e\right);{\left[-32,32\right]}^{n}$$

Here, using the Holm–Bonferroni method, it is shown how statistically different the μGA algorithm is from other optimization algorithms. The *Y_i_* values were utilized to calculate the cumulative normal distribution (*P*) values.

(25) $$Y_{i}=\frac{C_{i}-C_{0}}{\sqrt{\frac{N_{A\;}\left(N_{A}+1\right)}{2\times 10}}}$$

*h* = 0, the first hypothesis is accepted;
*h* = 1, the second hypothesis is accepted;
*C_i_* = (*i* shows the number of a compared algorithm) is calculated according to the rank score of each algorithm;
*R_i_* = rank score (*R_i_*), demonstrating the degree of performance of the algorithm;
*N_A_* = 2.

(26) $$\zeta_{i}=\frac{0.01}{N_{A}-i},\mu GA$$

(27) $$\zeta_{i}=\frac{0.05}{N_{A}-i},CGA$$

*P_i_* < $\zeta_{i}$ implies that the second hypothesis is accepted (*h* = 1). Otherwise, the first hypothesis is accepted (*h* = 0).

**Figure 15:**
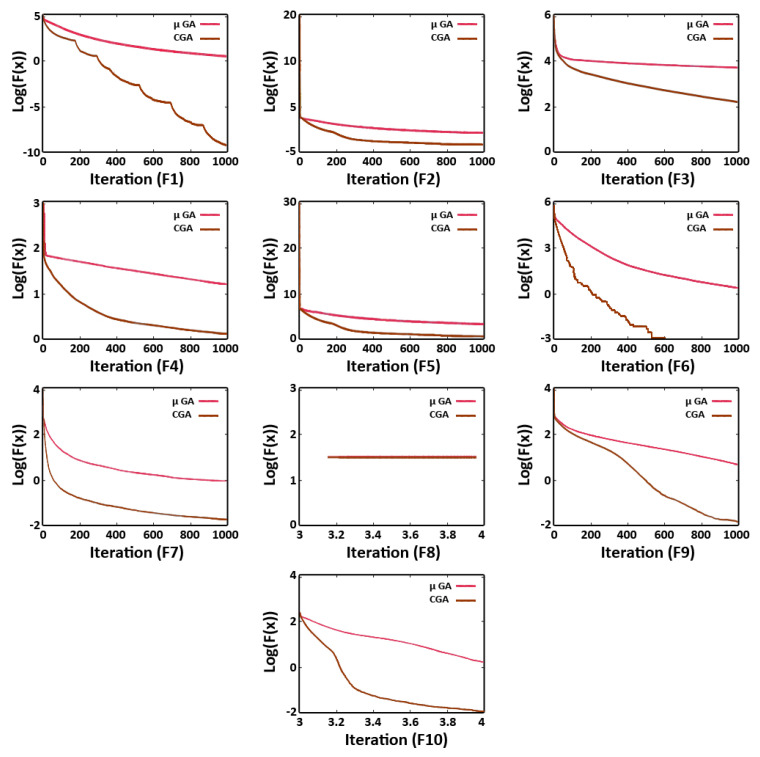
Convergence graphs of the benchmark functions.

We performed the benchmark test functions and utilized the Holm–Bonferroni statistical test results to justify the performance of the proposed algorithm. Table 9 represents the benchmark functions’ results, while the convergence graphs are depicted in Figure 15, and the Holm–Bonferroni statistical test results are shown in Table 10. The performance comparison between the CGA and μGA was analyzed by using a statistical test. From the results, the μGA algorithm showed the best performance; thus, this algorithm was assigned the first rank, whereas the CGA was assigned the second rank.

#### 3.2.4. Authentication of the Achieved Final Coordinates

For the authentication of the achieved final coordinates of the RSS_max_ of the proposed scheme, we utilized Equation (1) w/o employing the CGA and μGA for both Locations 1 and 2. Table 11 shows the other coordinates we verified; we can observe from the table that the RSS_max_ showing the final coordinates of CGA for the user position 1 (0.8, 0.7, 0.2 = −19.6 dBm) and user position 2 (0.9, 0.9, 0.08 = −14.7 dBm); and of μGA for the user position 1 (1.5, 1.9, 0.4 = −17.9 dBm) and user position 2 (1.3, 1.0, 0.1 = −12.1 dBm) had a numerical value that was similar to that obtained through the optimization using the CGA and μGA.

## 4. Conclusions and Future Works

A novel optimized V-VLC receiver design was introduced, and its experimental evaluation was performed in the NLOS VLC scenario to optimize the received power for vehicular communications. The proposed scheme provides a competent computation for the user to redirect the device in the direction of the best alignment to achieve the RSS_max_. The proposed V-VLC receiver was designed for automotive applications. The results demonstrated that the proposed system is suitable for the envisioned automotive applications, with good BER values even if the SNR is low.

Considering the real dynamic, long distances and the environmental factors, the proposed receiver could be an optimized solution that could aid in establishing communication with different data rates depending on the SNR. Moreover, the presented system could assist the driver in real conditions by providing an optimal communication quality and the maximum RSS. The μGA’s result showed that the RSS_max_ was improved by −1.7 dBm and −2.6 dBm (for Location 1 and Location 2), which proved that μGA is more efficient. Even though the results showed that the μGA improved the result, there was still the opportunity for the convergence of the optimal solutions by retaining unique fitness values in each generation.

However, the proposed method has some limitations. Although the proposed method utilizing the μGA proved to be a fast problem-solving approach, the random convergence of the solutions in a variant problem as regards the fitness function caused problems. Besides, the wrong choice of the fitness function may lead to critical problems; it could be unable to find a solution. Another concern is the early convergence of the μGA, which should be reconsidered at the time of the solution. Therefore, to mitigate these problems, in the future, a brief study of the hierarchical scheme with real and binary mutation operators could be carried out to expand the application of the proposed method to multi-objective optimization problems with large-scale decision variables. We could integrate the μGA with other state-of-the-art metaheuristics. Furthermore, work can be performed to improve the proposed system’s performance in terms of noise mitigation and long-distance communication. Besides, the proposed V-VLC implementation technique can be investigated further, considering a variety of characteristics such as the TX, RX, and blockage position to direct the PD in real time for the best RSSmax. This research can be envisioned as a context-aware system that can be fully designed and implemented as an embedded system to solve real-time traffic issues.

## Figures and Tables

**Table 1:** Electrical and optical characteristics of the S7686.

Parameter	Typical Value	Unit
Spectral response range	480 to 660	nm
Peak sensitivity wavelength	550	nm
Photo sensitivity	0.38	A/W
Short circuit current	0.45 @ 100 lx	μA
Dark current	2	pA

**Table 2:** Output of the TIA, GC, and LPF.

Sensitivity	Control Signal		Resistive Combination	Measuring Range (lx)	Measuring Condition (lx)	Output Voltage (V)		
	S1	S2				I-V Output	Gain Amplifier Output	LPF Output
High	L	L	R_fh_	0–300	300	−2.4	−3.6	3.6
Medium	L	H	R_fh_ // R_fM_	300–1500	1500	−2.4	−3.6	3.6
Low	H	L	R_fH_// R_fL_	1500–7500	7500	−2.4	−3.6	3.6

**Table 3:** A review of the published state-of-the-art in VLC (in terms of the RSS).

Output Parameter	Reference	Receiver	Accuracy	Objective	Processing Time
	[40]	PD	8 cm	Indoor positioning	-
	[41]	PD	1.5 cm	Indoor positioning	-
	[42]	PD	0.3 cm–0.7 cm	Indoor positioning	-
RSS	[24]	PD	-	Achieve maximum RSS in indoor VLC	0.21 ms (GA)
	[25]	PD	-	Achieve maximum RSS in indoor VLC	30 ms (PSO)
	This work	PD	-	Achieve maximum RSS in outdoor VLC	μGA processing time 0.7 s

**Table 4:** Summary of the key simulation parameters of the NLOS environment.

Parameters	Values
No. of LEDs	3
LED Power	10 W
TX (1st) location (m)	[0, −1, 2]
TX (2nd) location (m)	[0, 1, 2]
TX (3rd) location (m)	[0, 1, −2]
RX Location 1/Coordinate *A*_1_ (m)	[1, 2, 0]
RX Location 1/Coordinate *A*_2_ (m)	[1, 1, 0]
Blockage location for *Rx* 1 (m)	[1.2, 2, 1.5]
Blockage location for *Rx* 2 (m)	[1.5, 1, 1]
Lambertian angle in degrees	60^∘^
FOV of user (PD)	Ψ_c_ = 20Π180^∘^
PD area	0.01 × 0.01 m^2^

**Table 5:** Key simulation parameters used for the CGA.

Symbol	Parameters	Values
N_pop_	Population size	50
Iter_max_	Maximum iteration	300
P_crossover_	Probability of crossover	0.5
P_mutation_	Probability of mutation	0.05
Execution time	Time (seconds)	7.1

**Table 6:** Key simulation parameters used for the μGA.

Symbol	Parameters	Values
N_pop_	Population size	50
Iter_max_	Maximum iteration	300
P_crossover_	Probability of crossover	0.25
P_mutation_	Probability of mutation	0.01
Execution time	Time (seconds)	0.7

**Table 7:** Alignment of finding the maximum RSS.

Algorithm Name	*X* (m)	*Y* (m)	*Z* (m)
CGA	0.8	0.7	0.2
μGA	1.5	1.9	0.4

**Table 8:** Finding the alignment of the maximum RSS.

Algorithm Name	*X* (m)	*Y* (m)	*Z* (m)
CGA	0.9	0.8	0.08
μGA	1.3	1.0	0.1

**Table 9:** Benchmark functions’ results.

Function	Mean Value/Standard Deviation (CGA)	Mean Value/Standard Deviation (μGA)	Rank CGA	Rank μGA
1	2.43 × 10^0^/8.45 × 10^−1^	2.12 × 10^−9^/3.44 × 10^−9^	6	1
2	5.10 × 10^−1^/1.05 × 10^−1^	1.21 × 10^−2^/1.11 × 10^−2^	6	2
3	1.19 × 10^4^/3.49 × 10^3^	1.03 × 10^3^/4.83 × 10^2^	6	3
4	1.12 × 10^1^/2.80 × 10^1^	1.22 × 10^0^/1.90 × 10^−1^	6	1
5	5.04 × 10^2^/2.44 × 10^2^	1.00 × 10^2^/5.55 × 10^1^	6	1
6	3.72 × 10^0^/1.75 × 10^0^	0/0	4	1
7	2.04 × 10^−1^/4.30 × 10^−2^	1.34 × 10^−2^/3.91 × 10^−3^	6	1
8	−1.51 × 10^4^/4.05 × 10^2^	−2.19 × 10^4^/2.00 × 10^−1^	2	1
9	4.48 × 10^0^/2.03 × 10^0^	2.18 × 10^−2^/2.83 × 10^−2^	2	1
10	3.52 × 10^−1^/1.14 × 10^−1^	7.95 × 10^−3^/6.05 × 10^−3^	2	1
-	-	-	Avg.rank 4.6 Rank 2	Avg.rank 1.3 Rank 1

**Table 10:** Holm–Bonferroni statistical test results.

Algorithm Name	Score	*Z*	*P*	ζ	*h*
CGA	4.6000	−3.2696	0.00051	0.0081	1 (accepted)
μGA	7.7034	-	-	-	-

**Table 11:** Verification of the achieved final coordinates without using the optimization algorithm.

Algorithm Name	User Position 1 (m)	Signal Power Received (dBm) User 1	User Position 2 (m)	Signal Power Received (dBm) User 2
w/o CGA	X, Y, Z 1.13, 2.87, 0.08 0.25, 2.10, 0.07 **0.8, 0.7, 0.2** 0.5, 0.25, 0.3	−20.98 −21.08 **−19.6** −25.70	X, Y, Z 0.13, 1.87, 0.01 1.25, 2.10, 0.05 **0.9, 0.9, 0.08** 2.5, 0.2, 0.3	−25.40 −19.08 **−14.7** −16.70
w/o μ GA	X, Y, Z 1.15, 0.37, 0.03 1.25, 1.02, 0.01 **1.5, 1.9, 0.4** 0.6, 0.5, 0.23	−26.40 −19.08 **−17.9** −23.70	X, Y, Z 1.13, 2.87, 0.08 0.25, 2.10, 0.07 **1.3, 1.0, 0.1** 0.5, 0.25, 0.3	−14.40 −18.08 **−12.1** −15.70

## Data Availability

Not applicable.
